# Binder-free rGO–Si composite anodes with controlled silicon content and composition-dependent electrochemical performance

**DOI:** 10.1039/d6ra01135g

**Published:** 2026-04-08

**Authors:** Pham The Tan, Nguyen Van Tu, Nguyen Van Hao

**Affiliations:** a Hung Yen University of Technology and Education Viet Tien ward Hung Yen Province Vietnam; b Institute of Materials Science, Vietnam Academy of Science and Technology Hanoi Vietnam; c TNU-University of Sciences Phan Dinh Phung ward Thai Nguyen Province Vietnam haonv@tnus.edu.vn

## Abstract

Silicon is a promising anode material for lithium-ion batteries due to its extremely high theoretical capacity, yet its practical implementation is hindered by severe volume expansion and interfacial instability during cycling. In this work, binder-free reduced graphene oxide-silicon (rGO–Si) composite anodes with systematically controlled silicon contents (25–75 wt%) are fabricated to elucidate the role of composition in governing electrochemical behavior. The rGO framework forms a continuous conductive network and a mechanically compliant matrix, facilitating more uniform silicon dispersion and buffering volume changes. Electrochemical measurements reveal that the rGO–Si composite containing 75 wt% silicon delivers the best overall electrochemical performance among the investigated compositions, achieving a reversible capacity of ∼1150 mA h g^−1^ at 0.5 A g^−1^ with ∼90% capacity retention after 100 cycles. Thermogravimetric analysis confirms the compositional robustness and enhanced thermal stability of the composite structure, while electrochemical impedance spectroscopy demonstrates reduced charge-transfer resistance and improved interfacial kinetics compared with pristine silicon. These results highlight the critical role of compositional optimization in rGO–Si composite anodes and provide a practical strategy for developing durable and high-performance silicon-based anodes for next-generation lithium-ion batteries.

## Abstract 2

Binder-free rGO–Si anodes: composition-controlled performance optimization.

## Graphical abstract

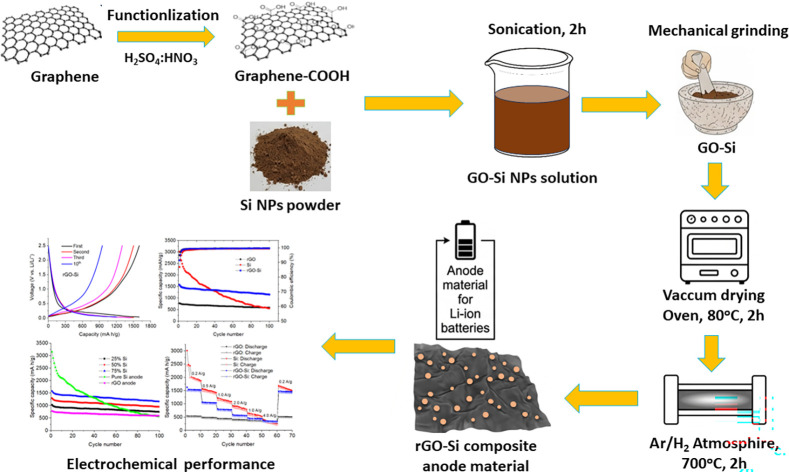

## 1. Introduction

Lithium-ion batteries (LIBs) have become the dominant energy storage technology for portable electronics, electric vehicles, and grid-scale applications, owing to their favorable balance between energy density, cycle life, and operational reliability.^1–3^ In recent years, the continuous demand for higher energy density LIBs, particularly driven by electric mobility and renewable energy integration, has imposed increasing pressure on electrode materials to surpass the intrinsic limitations of conventional designs.^4^ Among the electrode components, the anode represents a key bottleneck for further increasing the energy density of LIBs, as commercial graphite is fundamentally limited by its low theoretical capacity (∼372 mA h g^−1^).^2,4–6^ This limitation has stimulated extensive research into alternative anode materials with substantially higher lithium storage capability.

Silicon has emerged as a highly attractive anode candidate due to its exceptionally high theoretical specific capacity (∼3579 mA h g^−1^), appropriate lithiation potential, natural abundance, and environmental compatibility, offering a viable pathway to substantially enhance both gravimetric and volumetric energy density of LIBs. Recent reviews have summarized the rapid progress and persistent challenges associated with silicon anodes for next-generation lithium batteries.^6–10^ However, its practical application remains severely hindered by intrinsic material challenges. The most critical issue arises from the enormous volume expansion of silicon during lithiation, often exceeding 300%, which generates substantial mechanical stress that leads to particle pulverization, structural degradation of the electrode, and loss of electrical contact within the electrode structure.^11–13^ These structural instabilities are further accompanied by continuous rupture and regeneration of the solid-electrolyte interphase (SEI), leading to low coulombic efficiency and rapid capacity decay.^12,14^ Additionally, the relatively low electrical conductivity of silicon further aggravates polarization and charge-transfer limitations, particularly under high current densities.^15^

To mitigate these issues, a wide range of material engineering strategies have been explored, including nanostructuring silicon and constructing silicon–carbon composite architectures designed to buffer volume expansion and improve electrical conductivity.^16–19^ Reducing silicon to nanoscale dimensions can partially alleviate mechanical stress and shorten lithium-ion diffusion pathways; however, these advantages are often accompanied by increased surface reactivity and interfacial instability.^11,17^ However, such nanostructured silicon still tends to exhibit high surface reactivity and unstable SEI formation, which compromises long-term cycling stability, especially at practical mass loadings.^18^ Consequently, integrating silicon with conductive carbon matrices has become a widely adopted approach to improve both mechanical resilience and electronic transport properties of the electrode.

Among various carbonaceous materials, graphene and reduced graphene oxide (rGO) have attracted particular interest due to their high electrical conductivity, mechanical flexibility, and tunable surface chemistry, making them promising matrices for silicon-based composite anodes.^5,20–23^ When incorporated into silicon-based composites, rGO is expected to form a continuous conductive network that facilitates efficient electron transport while simultaneously buffering the mechanical strain induced by silicon volume expansion.^24–26^ In addition, the two-dimensional morphology of rGO facilitates intimate interfacial contact with silicon particles, which is beneficial for maintaining electrode integrity during repeated lithiation/delithiation cycles.^26^ As a result, rGO–Si composite anodes have demonstrated improved cycling stability and rate performance compared to pristine silicon electrodes, as reported in several recent studies on graphene–silicon hybrid structures.^27–30^

Despite these advances, several critical challenges remain unresolved from a materials engineering standpoint. First, many reported rGO–Si electrodes rely on polymeric binders and conductive additives, which introduce electrochemically inactive components and may weaken the structural cohesion of the electrode during prolonged cycling.^14,28^ Second, the electrochemical behavior of rGO–Si composites is strongly influenced by the relative fraction of silicon and graphene within the electrode architecture. Excessive silicon loading can overwhelm the buffering capability of the carbon matrix, leading to mechanical degradation, whereas insufficient silicon content limits the achievable capacity enhancement.^29^ Although numerous rGO–Si composite architectures have been reported, most studies primarily emphasize structural design or performance optimization, while the fundamental relationship between silicon fraction, conductive network integrity, and charge-transfer kinetics in binder-free graphene frameworks remains insufficiently clarified.^10,19^

In contrast to many previously reported rGO–Si composite electrodes that rely on complex nanostructure engineering or polymeric binder systems, the present work focuses on a binder-free rGO framework in which the silicon fraction is systematically varied under identical fabrication conditions. This strategy enables the intrinsic role of silicon content on the integrity of the graphene conductive network, charge-transfer kinetics, and electrochemical stability to be directly evaluated without interference from additional structural variables.

From a mechanistic perspective, the electrochemical performance of binder-free rGO–Si composite electrodes is governed by the interplay between several structural and transport parameters, including the integrity of the conductive graphene network, the spatial distribution of silicon particles, and the charge-transfer resistance at the electrode–electrolyte interface.^10^ Variations in silicon fraction can significantly influence these parameters by modifying the connectivity of the conductive network, the degree of silicon aggregation, and the effective lithium-ion diffusion pathways within the composite structure. Consequently, understanding how the balance between active silicon loading and graphene network integrity affects electrode kinetics and structural stability is essential for the rational design of high-performance silicon-based anodes.

In light of these considerations, a clear understanding of how silicon content governs the structural integrity and electrochemical behavior of rGO–Si composite anodes remains essential. In this work, rGO–Si composites with systematically varied silicon fractions are designed and fabricated using a simple binder-free approach. By maintaining identical preparation conditions while varying the silicon fraction, the influence of electrode composition on microstructure, charge-transfer behavior, and electrochemical performance can be directly evaluated. The results reveal that the electrochemical performance does not increase monotonically with silicon content; instead, a more favorable composition emerges from the balance between the conductive rGO framework and the active silicon phase. The results provide composition-dependent insights into the structural and kinetic factors governing binder-free rGO–Si anodes, offering practical guidance for the rational design of high-performance lithium-ion battery anodes.

## 2. Experimental

### 2.1. Materials

Natural graphite powder (≥99.5 wt%) was used as the precursor for graphene oxide (GO) synthesis *via* a modified Hummers' method. Nano-sized silicon powder (average particle size: 50–100 nm, ≥99.9 wt%, Sigma-Aldrich) was employed as the silicon source.

All chemical reagents, including KMnO_4_, concentrated H_2_SO_4_, HNO_3_, H_2_O_2_, HCl, and ethanol, were of analytical grade and used as received. Deionized water (18.2 MΩ cm) was used throughout the synthesis process. Copper foil (∼10 µm) served as the current collector for electrode fabrication without the use of polymer binders or conductive additives.

For electrochemical evaluation, lithium metal foil was employed as the counter and reference electrode, and a microporous polypropylene separator was used. The electrolyte consisted of 1 M LiPF_6_ dissolved in ethylene carbonate and diethyl carbonate (EC/DEC = 1 : 1, v/v).

### 2.2. Synthesis of rGO–Si composite anodes

**Fig. 1:**
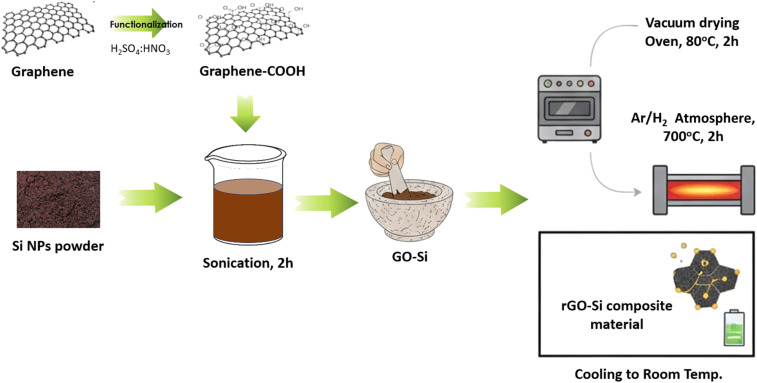
Schematic illustration of the preparation process for rGO–Si composite anodes, including graphene oxide synthesis, silicon incorporation, and thermal reduction.

A schematic illustration of the preparation process for the rGO–Si composite anodes is shown in Fig. 1. Graphene oxide obtained *via* the modified Hummers' method was dispersed in deionized water under mild ultrasonication to form a homogeneous suspension. Nano-sized silicon powder was subsequently introduced into the GO dispersion at predetermined mass ratios to obtain rGO–Si composites with different silicon contents. The mixture was mechanically stirred and then dried in a controlled manner to ensure uniform distribution of silicon particles within the graphene oxide matrix.

Subsequently, the dried precursor was thermally treated under an inert atmosphere to induce the reduction of GO to reduced graphene oxide (rGO) and to facilitate intimate contact between rGO sheets and silicon nanoparticles. This process resulted in a self-supporting rGO–Si composite structure, in which the conductive rGO network served as both an electronic pathway and a structural buffer to accommodate silicon volume changes during electrochemical cycling.

### 2.3. Electrode fabrication and cell assembly

The as-prepared rGO–Si composite was directly used as the anode active material without the addition of polymer binders or conductive additives. The composite material was uniformly deposited onto copper foil current collectors and subsequently dried to obtain free-standing electrode layers with controlled mass loading. The average mass loading of the active material was approximately 1.5 mg cm^−2^. This fabrication strategy avoids the use of polymer binders and conductive additives, thereby increasing the proportion of electrochemically active material.

CR2032-type coin cells were assembled in an argon-filled glovebox with oxygen and moisture levels below 0.1 ppm. Lithium metal foil was employed as the counter and reference electrode. A microporous polypropylene separator was used to electrically isolate the electrodes, and a liquid electrolyte consisting of 1 M LiPF_6_ dissolved in ethylene carbonate and diethyl carbonate (EC/DEC = 1 : 1, v/v) was introduced into the cells. All electrochemical measurements were conducted at room temperature.

### 2.4. Materials characterization

The structural and morphological features of the synthesized materials were characterized using advanced electron microscopy techniques. Field-emission scanning electron microscopy (FE-SEM, Hitachi S-4800, Japan) operated at 5 kV was employed to examine surface morphology. The elemental distribution and compositional uniformity were further evaluated through energy-dispersive X-ray spectroscopy (EDS) mapping conducted on a scanning electron microscope (JSM-IT800, JEOL, Japan).

Crystalline structures and phase characteristics were identified using X-ray diffraction (XRD, Bruker D2 Phaser) with Cu Kα radiation. Raman spectroscopy (XploRA PLUS, 532 nm excitation) was applied to probe the structural evolution of graphene oxide during reduction and its interaction with silicon. In addition, Fourier transform infrared (FTIR) spectroscopy (Jasco-4600) was used to analyze surface functional groups, providing complementary information on chemical bonding within the composite. Thermogravimetric analysis (TGA) was conducted to evaluate the thermal stability and compositional characteristics of Si, rGO, and rGO–Si composite materials. The measurements were performed using a thermogravimetric analyzer under an air atmosphere with a heating rate of 5 °C min^−1^ over a temperature range from room temperature to 800 °C.

### 2.5. Electrochemical measurements

The electrochemical performance of the prepared electrodes was evaluated using CR2032-type coin cells assembled in an argon-filled glovebox. Cyclic voltammetry (CV) measurements were conducted within a voltage window of 0.01–2.5 V (*vs.* Li/Li^+^) to investigate the redox behavior and electrochemical reversibility of the electrodes. Galvanostatic charge–discharge (GCD) tests were performed over the same potential range at various current densities to determine specific capacity, cycling stability, and rate capability.

Long-term cycling performance and coulombic efficiency were assessed under constant current conditions, while rate capability was evaluated by stepwise variation of the applied current density. All electrochemical measurements were carried out at ambient temperature, and the reported capacities were calculated based on the mass of active material in the electrodes.

Electrochemical impedance spectroscopy (EIS) measurements were carried out using a potentiostat/galvanostat to investigate the interfacial charge-transfer behavior and lithium-ion transport kinetics of the electrodes. The impedance spectra were recorded in the frequency range of 100 kHz to 0.01 Hz with an AC perturbation amplitude of 5 mV at open-circuit potential. EIS measurements were performed for fresh cells before cycling and after prolonged cycling to evaluate the evolution of interfacial resistance.

## 3. Results and discussion

### 3.1. Structural analysis

**Fig. 2:**
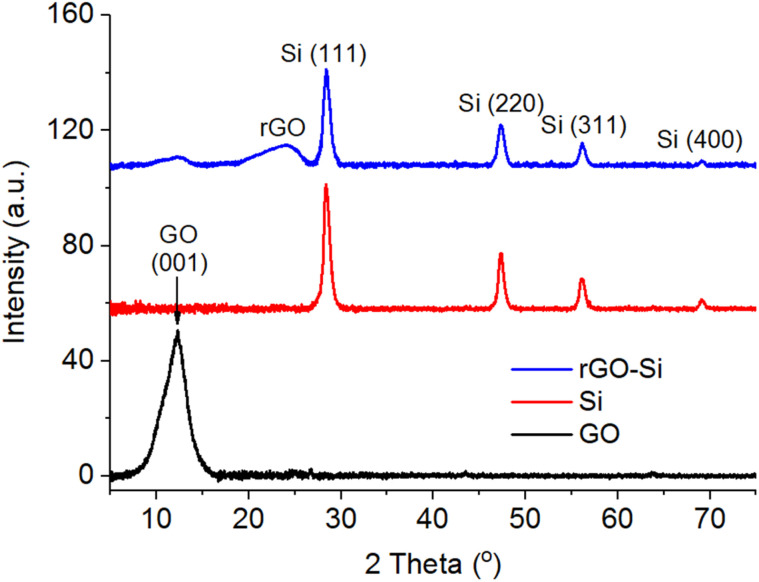
X-ray diffraction patterns of GO, Si and rGO–Si materials.

Fig. 2 presents the X-ray diffraction (XRD) patterns of graphene oxide (GO), pristine silicon nanoparticles, and the rGO–Si composite. The diffraction pattern of GO exhibits a distinct peak centered at 2*θ* ≈ 10–11°, which is characteristic of the (001) reflection arising from the enlarged interlayer spacing induced by abundant oxygen-containing functional groups introduced during the oxidation process.^31,32^ The presence of this peak is consistent with the successful oxidation of graphite and the formation of a layered GO structure with expanded galleries, which facilitates subsequent incorporation of silicon nanoparticles, as further evidenced by the uniform dispersion observed in SEM analysis.

In contrast, pristine silicon displays a series of sharp and well-defined diffraction peaks located at approximately at 2*θ* ≈ 28.4°, 47.3°, 56.1°, and 69.1°, which can be indexed to the (111), (220), (311), and (400) planes of crystalline silicon with a diamond cubic structure (JCPDS no. 27-1402). The narrow peak widths indicate a high degree of crystallinity, providing a structurally well-defined host for reversible Li–Si alloying reactions during electrochemical cycling.^33,34^

For the rGO–Si composite, the disappearance of the GO (001) reflection together with the emergence of a broad diffraction feature in the range of 2*θ* ≈ 24–26° suggests the effective reduction of GO and partial restoration of graphitic stacking. Meanwhile, the characteristic diffraction peaks of crystalline silicon remain clearly discernible without noticeable peak shifting or broadening, indicating that the composite formation process does not induce detectable lattice distortion or amorphization of silicon, even at relatively high silicon loading. This observation suggests that the reduction and composite assembly processes preserve the intrinsic crystal structure of silicon nanoparticles.

Importantly, no additional diffraction peaks associated with crystalline impurity phases or silicon oxide species are detectable within the resolution of XRD, confirming that the adopted synthesis route maintains high phase purity of the composite. Similar structural features have been reported for graphene–silicon hybrid anodes, where reduced graphene oxide functions as a conductive and mechanically resilient matrix while retaining the crystallinity of silicon.^33,34^ The coexistence of crystalline Si and a partially restored rGO framework establishes a structurally integrated architecture that is expected to facilitate efficient electron transport while accommodating the large volume variation of silicon during lithiation and delithiation processes, thereby providing a favorable structural foundation for the enhanced electrochemical performance discussed in subsequent sections.

### 3.2. Chemical bonding and structural disorder analysis

**Fig. 3:**
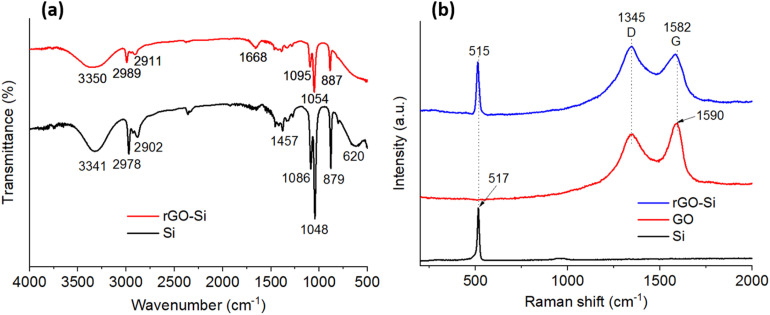
(a) FTIR spectra of Si and rGO–Si; (b) Raman spectra of Si, GO and rGO–Si material.

Fig. 3a shows the Fourier-transform infrared (FTIR) spectra of pristine silicon nanoparticles and the rGO–Si composite, providing insights into surface functional groups and interfacial chemical features. For pristine silicon, the absorption band observed at approximately 3340 cm^−1^ can be attributed to O–H stretching vibrations associated with surface hydroxyl groups or adsorbed moisture, which are commonly present on nano-sized silicon exposed to ambient conditions. The weak bands appearing in the region of 2970–2900 cm^−1^ are assigned to residual C–H stretching vibrations, likely originating from trace organic species adsorbed on the silicon surface. In addition, the bands located at around 1080–1050 cm^−1^ and 880–620 cm^−1^ correspond to Si–O–Si stretching and bending modes, indicating the presence of a thin native oxide layer on the silicon nanoparticles, which is frequently reported for silicon-based anode materials.^35,36^

In contrast, the FTIR spectrum of the rGO–Si composite exhibits several characteristic features associated with reduced graphene oxide. The broad absorption band centered near 3350 cm^−1^ is attributed to O–H stretching vibrations, reflecting the partial retention of hydroxyl groups after the reduction process. The peaks observed at approximately 2910 and 2985 cm^−1^ correspond to C–H stretching modes, while the band located at around 1665–1670 cm^−1^ is assigned to C=C skeletal vibrations, suggesting partial restoration of sp^2^ – hybridized carbon domains. Furthermore, the absorption bands in the range of 1090–1050 cm^−1^ are associated with C–O stretching vibrations, indicating that a certain amount of oxygen-containing functional groups remains on the rGO sheets.^32,37,38^

Compared with typical GO spectra reported in the literature, the reduced intensity of oxygen-related bands in the rGO–Si composite suggests effective but incomplete reduction of GO. Such controlled retention of surface functionalities is considered beneficial, as it can enhance interfacial interactions between rGO sheets and silicon nanoparticles, thereby contributing to improved structural stability of the composite electrode during electrochemical cycling.^39^

Fig. 3b presents the Raman spectra of pristine Si, GO, and the rGO–Si composite. The Raman spectrum of pristine silicon exhibits a sharp and intense peak at approximately 515–517 cm^−1^, corresponding to the first-order transverse optical (TO) phonon mode of crystalline silicon. The narrow linewidth indicates high crystallinity of the silicon nanoparticles, which is consistent with the XRD results discussed previously.^40^

The GO sample shows two prominent bands at around 1345 cm^−1^ (D band) and 1590 cm^−1^ (G band). The D band originates from defect-induced breathing modes of sp^2^ carbon rings, whereas the G band corresponds to the in-plane stretching of sp^2^ – hybridized carbon atoms. The relatively high D-band intensity reflects the high defect density and abundant oxygen functional groups introduced during the oxidation process.^41,42^

For the rGO–Si composite, the D and G bands are observed at approximately 1345 cm^−1^ and 1582 cm^−1^, respectively. Notably, the intensity ratio of the D band to the G band (*I*_D_/*I*_G_) increases compared to that of GO, which is commonly attributed to the formation of smaller sp^2^ domains and the introduction of new defect sites during the reduction of GO. This behavior is widely reported for reduced graphene oxide and is indicative of partial restoration of graphitic structures accompanied by increased structural disorder. This change suggests the formation of smaller sp^2^ domains and a higher degree of structural disorder as a result of GO reduction. Such behavior is reflecting partial restoration of sp^2^ domains accompanied by defect redistribution during GO reduction.^43–45^ In addition, the characteristic Si phonon peak remains discernible in the composite spectrum, confirming the coexistence of crystalline silicon and rGO within the hybrid material.

The simultaneous presence of defect-rich rGO and crystalline silicon in the composite highlights the successful integration of the two components at the structural level. The rGO framework provides a conductive and flexible matrix, while residual functional groups facilitate interfacial contact with silicon nanoparticles. Such an interfacial architecture is expected to promote efficient charge transport, accommodate mechanical stress arising from silicon volume changes, and contribute to the improved electrochemical performance of the rGO–Si composite anode, as further discussed in the subsequent electrochemical analyses.^3,46,47^

### 3.3. Morphological, elemental, thermal stability and compositional analysis

**Fig. 4:**
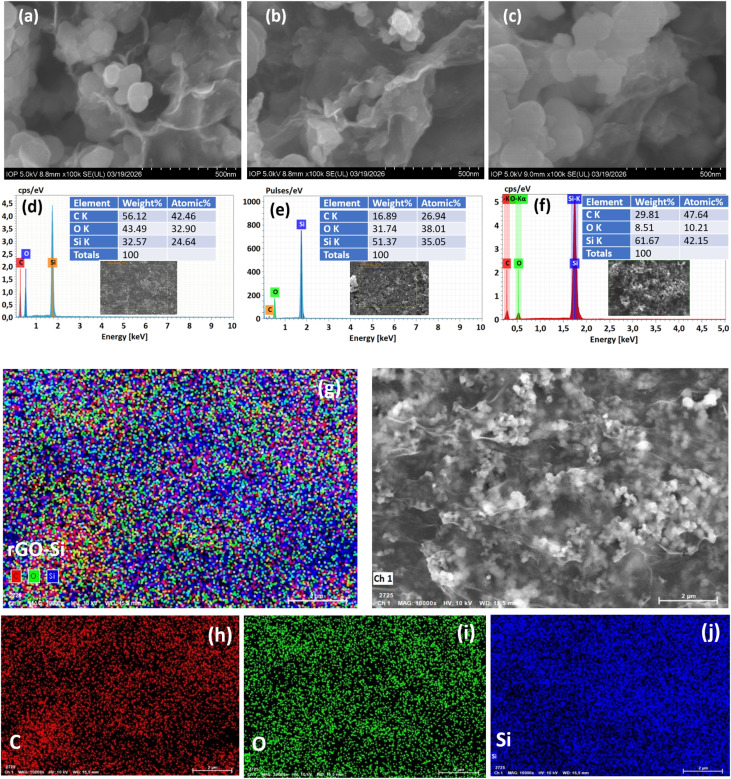
SEM images of rGO–Si composites with different Si contents: (a) 25 wt%, (b) 50 wt%, and (c) 75 wt% (all at identical magnification). Corresponding EDX spectra are shown in (d–f). (g) Elemental mapping of the rGO–Si composite (overlay), and (h–j) corresponding distribution maps of C, O, and Si, respectively, demonstrating the homogeneous distribution of elements within the composite.

Fig. 4 presents the surface morphology and elemental characteristics of the rGO–Si composites with different silicon contents, as characterized by scanning electron microscopy (SEM) and energy-dispersive X-ray spectroscopy (EDX). The SEM images (Fig. 4a–c), acquired at identical magnification, enable a direct comparison of the morphological evolution as a function of Si content. All samples exhibit a crumpled and wrinkled structure typical of reduced graphene oxide, forming a continuous and interconnected network. Silicon nanoparticles are distributed within this framework; however, they do not form a perfectly uniform or fully encapsulated structure, but rather appear partially embedded within the rGO sheets. Additional SEM images at different magnifications are provided in the SI (Fig. S1–S3), further confirming the observed morphology and structural features.

As the silicon content increases from 25 wt% to 75 wt%, a clear change in morphology can be observed. The sample with 25 wt% Si (Fig. 4a) shows relatively dense stacking of graphene sheets with limited visible particle clustering, suggesting that rGO dominates the structural framework. At 50 wt% Si (Fig. 4b), the dispersion of silicon nanoparticles becomes more apparent, with improved spacing between graphene layers, indicating a more balanced composite structure. In contrast, the 75 wt% Si sample (Fig. 4c) exhibits increased particle aggregation and reduced structural uniformity, which may weaken the continuity of the conductive network. Such composition-dependent structural evolution is expected to influence both electron transport and mechanical stability during electrochemical cycling. The multi-scale SEM observations presented in the SI (Fig. S1–S3) further support this trend, revealing consistent structural features across different length scales.

The observed morphology suggests that the rGO framework plays a critical role in mitigating silicon agglomeration by spatially separating particles and providing a flexible matrix. The wrinkled and layered nature of rGO can offer internal void space to partially accommodate the volume expansion of silicon during lithiation, which is essential for improving the structural stability of silicon-based anodes.^46,48,49^ Moreover, the continuous rGO network is expected to serve as a conductive scaffold, facilitating electron transport throughout the electrode and promoting more homogeneous electrochemical reactions. At the same time, the mechanical flexibility of rGO sheets can buffer the stress induced by repeated volume changes of silicon, thereby mitigating particle fracture and preserving electrical connectivity during prolonged cycling.^50,51^ It should be noted that these functional roles are inferred from the observed structural features and are consistent with previously reported rGO–Si systems.

The elemental composition of the composites was further analyzed by EDX spectroscopy (Fig. 4d–f), which confirms the presence of carbon, silicon, and oxygen in all samples, consistent with the designed material system. It should be noted that EDX is a surface-sensitive technique and provides mainly qualitative or semi-quantitative information. The carbon signal originates from the rGO framework, while the oxygen signal can be attributed to residual oxygen-containing functional groups on partially reduced graphene oxide as well as the native oxide layer on silicon nanoparticles. The relative elemental contents obtained from EDX show deviations from the nominal composition, which can be attributed to the localized and surface-sensitive nature of the technique, especially in heterogeneous systems such as rGO–Si composites.

To provide a more representative evaluation of elemental distribution, EDX mapping was performed (Fig. 4g–j). The overlay image (Fig. 4g) and the corresponding elemental maps of C, O, and Si (Fig. 4h–j) reveal that all elements are homogeneously distributed and spatially interconnected across the observed area, without evidence of large-scale phase segregation. Although minor local variations and slight aggregation of silicon can be observed, the overall distribution confirms that silicon nanoparticles are effectively incorporated within the rGO framework. Such a relatively uniform dispersion is crucial for ensuring consistent electrochemical activity and minimizing localized stress accumulation during cycling.^52,53^ The structural characteristics of rGO and its reduction are further confirmed by Raman spectroscopy and XRD analysis, as discussed in the following section. Overall, the SEM observations together with EDX elemental analysis indicate that the adopted fabrication strategy enables the formation of a structurally integrated rGO–Si composite with favorable morphological and interfacial characteristics, which underpin the enhanced electrochemical performance discussed in the following sections.

**Fig. 5:**
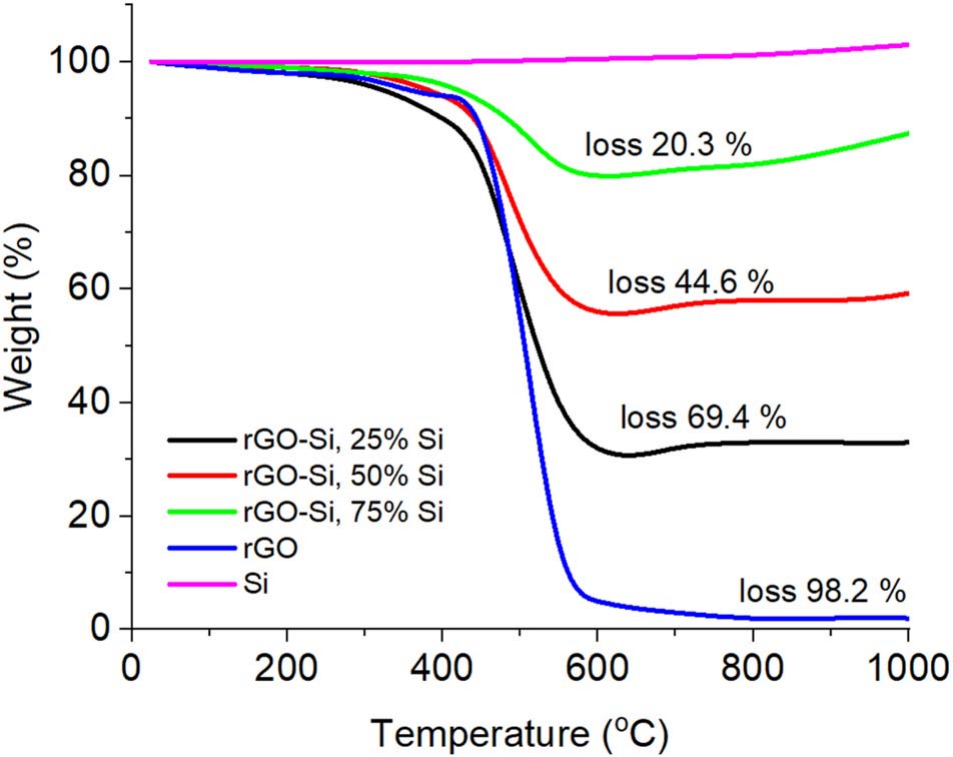
TGA curves of rGO, Si, and rGO–Si composites with different Si contents (25–75 wt%) recorded in air at 5 °C min^−1^.

Thermogravimetric analysis (TGA) was conducted to evaluate the thermal stability and to provide semi-quantitative confirmation of the composition of the rGO–Si composites prior to electrochemical testing (Fig. 5), as this technique is commonly employed for hybrid carbon–silicon anode materials. As shown in Fig. 5, pristine rGO exhibits a rapid mass loss at elevated temperatures, which is associated with oxidative decomposition of the carbon framework in air, consistent with the reported thermal behavior of reduced graphene-based materials.^54^

In contrast, pristine silicon shows negligible mass loss over the entire temperature range and a slight mass increase at high temperatures due to oxidation to form a stable SiO_2_ layer, reflecting the intrinsic thermal robustness of crystalline silicon.^55^ For the rGO–Si composites, the overall weight loss decreases systematically with increasing silicon content, indicating a reduced contribution from the thermally labile rGO component. The residual mass at high temperature correlates well with the nominal Si loading, confirming effective compositional control in the composite materials. Similar use of TGA to validate the composition of graphene–silicon hybrid anodes has been reported previously.^54^ It should be noted that TGA provides bulk compositional information, which complements the surface-sensitive EDX analysis. The good agreement between the residual mass and the nominal Si content suggests that the overall composition of the composite is well controlled, and that the relatively high oxygen signal observed in EDX is mainly associated with surface functional groups and native oxide layers rather than representing the bulk composition of the material. Importantly, the TGA results are consistent with the structural and morphological analyses discussed above (XRD, Raman, and SEM/EDX), collectively confirming the successful integration of crystalline silicon within the rGO framework. The enhanced thermal stability observed for Si-rich composites reflects improved compositional stability during thermal treatment and electrode processing, rather than direct electrochemical cycling.

### 3.4. Electrochemical behavior of rGO, Si, and rGO–Si anodes

**Fig. 6:**
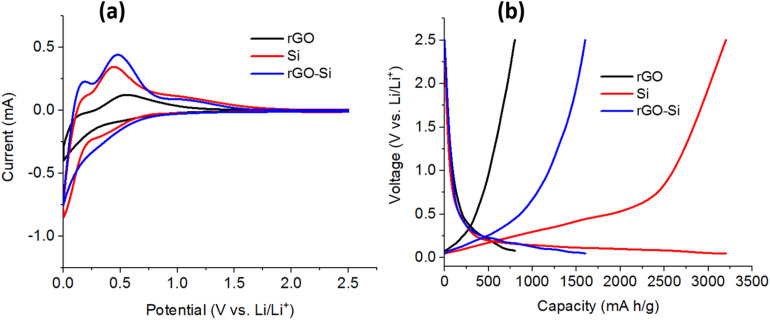
(a) Cyclic voltammetry (CV) curves and (b) galvanostatic charge–discharge profiles of rGO, Si and rGO–Si anodes measured in the voltage range of 0–2.5 V at room temperature at a current density of 0.5 A g^−1^.

The CV curves of the three electrodes recorded at a scan rate corresponding to the applied conditions are shown in Fig. 6a. The rGO electrode exhibits broad and featureless cathodic and anodic responses over the entire potential range, which are characteristic of surface-dominated lithium storage processes in disordered carbon materials. Such behavior is commonly attributed to a combination of electrical double-layer capacitance and pseudocapacitive lithium adsorption at defect sites and residual oxygen-containing functional groups on rGO sheets.^56,57^ The absence of sharp redox peaks indicates that lithium storage in rGO is largely non-alloying in nature. In contrast, the pristine silicon electrode displays distinct cathodic peaks at potentials below approximately 0.2 V during the reduction sweep, corresponding to the stepwise alloying of crystalline silicon with lithium to form amorphous LixSi phases. During the subsequent anodic sweep, broad oxidation peaks centered between ∼0.3 and 0.6 V are observed, which can be assigned to the dealloying process of LixSi back to amorphous silicon.^58,59^ These features reflect the typical lithiation–delithiation mechanism of silicon anodes, which is often accompanied by pronounced polarization and limited reversibility due to large volume changes and sluggish charge-transfer kinetics.

Notably, the rGO–Si composite electrode exhibits CV profiles that combine features from both constituents, with enhanced current response and more stable redox characteristics compared to pristine silicon. The cathodic and anodic peaks associated with silicon alloying and dealloying are still observable but appear broadened and slightly shifted, suggesting moderated reaction kinetics and reduced polarization. This behavior is consistent with the presence of a conductive rGO network that facilitates electron transport and improves interfacial charge transfer within the composite electrode.^60–62^ In addition, the larger enclosed area of the CV curve for rGO–Si relative to pure Si suggests increased electrochemically accessible active sites and more effective utilization of silicon within the composite architecture. The reproducibility of the CV behavior was verified using multiple independently assembled cells, which showed consistent electrochemical responses.

Fig. 6b presents the galvanostatic charge–discharge profiles of rGO, pristine Si, and rGO–Si anodes measured at a current density of 0.5 A g^−1^. The rGO electrode exhibits sloping voltage profiles without distinct plateaus during both lithiation and delithiation, which is typical for lithium storage governed by surface adsorption and pseudocapacitive processes in graphene-based materials.^57^ As a result, the reversible capacity of rGO remains relatively low despite its good cycling stability. The pristine silicon electrode delivers a very high initial discharge capacity exceeding 3000 mA h g^−1^, approaching the theoretical capacity of silicon under the applied testing conditions. It should be noted that the specific capacities reported for the rGO–Si electrode are calculated based on the total mass of the composite active material rather than normalized to the silicon fraction alone. Therefore, the practical capacity of the rGO–Si composite does not scale directly with the theoretical capacity of pure silicon, as only a portion of the silicon nanoparticles are electrochemically accessible within the composite architecture. This limited utilization is mainly attributed to restricted electronic pathways and incomplete electrolyte penetration within the composite structure. A pronounced voltage plateau below ∼0.2 V is observed during lithiation, corresponding to extensive Li–Si alloy formation. However, such deep lithiation is well known to induce severe volume expansion and mechanical stress, which often leads to rapid capacity fading upon subsequent cycling.^49,63^

In comparison, the rGO–Si composite anode demonstrates a more balanced electrochemical response. While maintaining a high reversible capacity, the voltage hysteresis between lithiation and delithiation is reduced relative to pristine silicon. Based on the electrode mass loading (∼1.5 mg cm^−2^), the corresponding areal capacity is in the range of ∼1.0–1.8 mA h cm^−2^ depending on the specific capacity. The areal capacity follows the same trend as the gravimetric capacity, indicating that the observed performance is primarily intrinsic to the material rather than influenced by low mass loading. The lithiation plateau appears less abrupt and extends over a broader potential range, indicating a more gradual and controlled alloying process. This moderated electrochemical behavior can be attributed to the mechanical confinement and buffering effect provided by the rGO framework, which constrains silicon expansion and helps preserve electrode integrity during repeated cycling.^64–66^ Furthermore, the rGO–Si electrode exhibits improved coulombic efficiency and reduced polarization compared to pure silicon, reflecting more stable interfacial reactions and enhanced electronic conductivity. The interconnected rGO network is expected to promote more uniform current distribution and mitigates localized over-lithiation of silicon particles, which is beneficial for suppressing premature degradation.^67,68^ Overall, the CV and charge–discharge results in Fig. 6 demonstrate that integrating silicon nanoparticles into an rGO matrix effectively reconciles high capacity with improved electrochemical reversibility, providing a solid basis for the enhanced cycling stability and rate capability discussed in the following sections.

### 3.5. Cycling behavior and long-term electrochemical stability

**Fig. 7:**
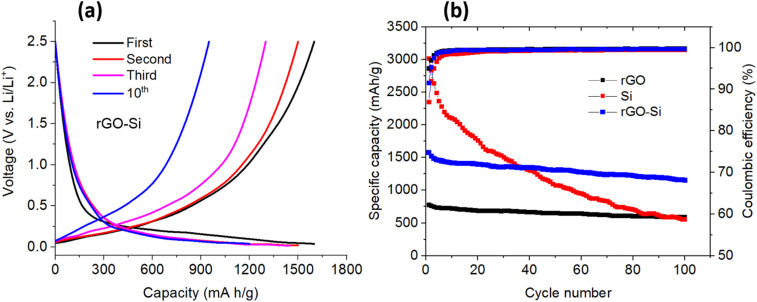
(a) Charge–discharge voltage profiles of the rGO–Si anode at the 1st, 2nd, 3rd, and 10th cycles charge and discharge profiles of rGO–Si anode material; (b) capacity retention profiles and corresponding coulombic efficiency of rGO, Si, and rGO–Si anode materials under a working voltage from 0 to 2.5 V at room temperature at a current density of 0.5 A g^−1^.

The long-term electrochemical stability of the rGO, pristine Si, and rGO–Si composite anodes was systematically evaluated by galvanostatic cycling at a current density of 0.5 A g^−1^, as presented in Fig. 7.

Fig. 7a displays the charge–discharge voltage profiles of the rGO–Si composite anode recorded at the 1st, 2nd, 3rd, and 10th cycles. During the initial lithiation process, the rGO–Si electrode delivers a high specific capacity accompanied by a sloping voltage region above ∼0.2 V followed by a more gradual plateau at lower potentials. The pronounced irreversible capacity loss observed in the first cycle can be mainly attributed to the formation of a solid electrolyte interphase (SEI) on both silicon nanoparticle surfaces and rGO sheets, as well as partial lithium trapping at defect sites within the rGO framework, phenomena commonly reported for silicon–carbon composite anodes.^69–71^

From the second cycle onward, the charge–discharge curves exhibit a high degree of overlap, indicating improved reversibility and stabilization of the electrode–electrolyte interface. A closer inspection of the curves (magnified view of Fig. 7a) confirms that the 2nd and 3rd cycles are highly similar but not identical, reflecting the stabilization of the electrode/electrolyte interface after the initial SEI formation, a behavior commonly reported for Si-based anodes. The reproducibility of this behavior was verified using multiple independently assembled cells, which exhibited consistent voltage profiles. The gradual convergence of the voltage profiles suggests that the rGO matrix helps accommodate the repeated volume changes of silicon during lithiation/delithiation, thereby mitigating progressive structural degradation. By the 10th cycle, the polarization between the charge and discharge curves becomes noticeably reduced compared to the initial cycle, reflecting enhanced charge-transfer kinetics and improved interfacial stability within the composite electrode.^72,73^ Importantly, no obvious abrupt voltage distortions or abnormal plateaus are observed upon cycling, implying that the rGO–Si architecture suppresses severe particle pulverization, a common failure mechanism in conventional silicon anodes.^74^

Fig. 7b compares the cycling performance and coulombic efficiency (CE) of rGO, pristine Si, and rGO–Si anodes over 100 cycles. The rGO electrode exhibits stable capacity retention throughout cycling but delivers a relatively low reversible capacity, consistent with lithium storage dominated by surface adsorption and limited intercalation processes in disordered carbon materials.^75^ In contrast, the pristine Si anode shows a high initial capacity exceeding 3000 mA h g^−1^, followed by rapid capacity fading upon prolonged cycling. This severe degradation is primarily associated with large volumetric expansion (>300%) of silicon during lithiation, leading to mechanical fracture, loss of electrical contact, and continuous SEI rupture and reformation.^2,76^ By comparison, the rGO–Si composite anode demonstrates a significantly improved balance between reversible capacity and cycling stability. Although its initial capacity is lower than that of pristine silicon, the rGO–Si electrode retains a substantially higher capacity after 100 cycles, indicating enhanced structural robustness and sustained electrochemical activity. The mitigated capacity decay highlights the role of the rGO framework in buffering silicon volume expansion and maintaining continuous electron transport pathways during repeated cycling.^77,78^

The initial coulombic efficiency is relatively low due to irreversible lithium consumption associated with SEI formation and side reactions. Furthermore, the coulombic efficiency of the rGO–Si anode increases rapidly after the initial activation cycles and stabilizes at values above ∼98% during prolonged cycling. This behavior indicate reduced parasitic side reactions and the formation of a relatively stable SEI layer. In contrast, the pristine Si electrode exhibits lower and more fluctuating CE values, reflecting ongoing interfacial degradation and continuous electrolyte consumption.^79,80^ Overall, the cycling and CE results demonstrate that integrating silicon nanoparticles into a conductive and mechanically compliant rGO matrix substantially enhances electrochemical durability while preserving high reversible capacity, thereby offering a viable strategy for improving the long-term performance of silicon-based anodes.

### 3.6. Effect of silicon content and rate capability performance

**Fig. 8:**
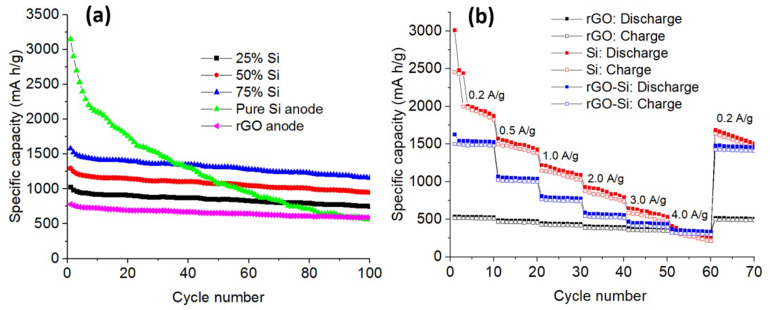
(a) Cycling performance of rGO–Si composite anodes with different silicon contents, together with rGO and pristine Si electrodes; (b) rate capability of rGO, Si, and rGO–Si anodes measured at current densities ranging from 0.2 to 4.0 A g^−1^.

The effect of silicon content on the cycling behavior of rGO–Si composite anodes was systematically investigated by comparing electrodes containing 25, 50, and 75 wt% Si, together with pristine rGO and pure Si anodes, as shown in Fig. 8a. The pure silicon anode delivers the highest initial specific capacity (>3000 mA h g^−1^); however, it undergoes rapid capacity decay during cycling, retaining only a small fraction of its initial capacity after 100 cycles. This pronounced fading behavior is characteristic of bulk silicon electrodes and is primarily associated with severe volume expansion during lithiation, leading to particle pulverization, loss of electrical contact, and continuous interfacial degradation.^58,81,82^ In contrast, the rGO anode exhibits excellent cycling stability but maintains a relatively low reversible capacity, reflecting its lithium storage mechanism dominated by surface adsorption and limited intercalation.^5^ The rGO–Si composite electrodes display intermediate and composition-dependent electrochemical behavior. At lower silicon contents (25 and 50 wt%), the composites show improved cycling stability compared to pure silicon, owing to the effective mechanical buffering provided by the rGO framework. However, the overall reversible capacity remains limited by the reduced fraction of electrochemically active silicon. This is also associated with the partial electrochemical utilization of silicon within the composite structure.

Among the investigated compositions, the rGO–Si electrode containing 75 wt% silicon exhibits the best overall electrochemical performance, combining high reversible capacity with sustained capacity retention over prolonged cycling. Notably, its retained capacity after extended cycling exceeds that of the pure silicon electrode, despite the latter's higher initial capacity. These results suggest that an appropriate rGO-to-Si ratio is important for achieving simultaneous enhancement of capacity and cycling stability. Within the investigated composition range, increasing the silicon content to 75 wt% provides an optimal balance between active material utilization and structural stability. Further improvement in performance is therefore governed not simply by increasing silicon fraction, but by maintaining an effective conductive and mechanically resilient rGO framework. The observed composition-dependent trend is consistent with previous studies on silicon–carbon composite anodes, which emphasize the importance of compositional optimization rather than maximizing silicon content alone.^7,68,77^

Fig. 8b compares the rate capability of rGO, pristine Si, and rGO–Si composite anodes evaluated at progressively increasing current densities from 0.2 to 4.0 A g^−1^, followed by a return to 0.2 A g^−1^. At low current density, the pristine Si electrode delivers a high specific capacity but exhibits a pronounced decline in capacity as the current density increases. This poor rate performance is mainly attributed to sluggish lithium-ion diffusion and increased charge-transfer resistance arising from structural degradation and fractured silicon particles under high-rate conditions.^83,84^ The rGO electrode shows relatively stable but modest capacities across the entire range of current densities, consistent with its fast surface-controlled kinetics but limited lithium storage sites.^85^ In contrast, the rGO–Si composite anode demonstrates improved rate performance, maintaining higher capacities than both individual components across the tested current density range. Even at a high current density of 4.0 A g^−1^, the rGO–Si electrode retains a considerable fraction of its low-rate capacity, highlighting its favorable kinetic characteristics.

When the current density is subsequently returned to 0.2 A g^−1^, the rGO–Si anode exhibits substantial recovery of its reversible capacity, indicating that the capacity loss at high rates is largely governed by kinetic limitations rather than irreversible structural damage. This behavior reflects the structural resilience of the composite architecture. The enhanced rate capability can be attributed to the interconnected rGO network, which is expected to provide continuous electron transport pathways, facilitate lithium-ion diffusion, and mitigates mechanical stress during rapid lithiation/delithiation processes.^61,80,86^ To further clarify how these kinetic advantages are manifested at the electrode–electrolyte interface, electrochemical impedance spectroscopy analysis was subsequently conducted.

### 3.7. Electrochemical impedance spectroscopy analysis

**Fig. 9:**
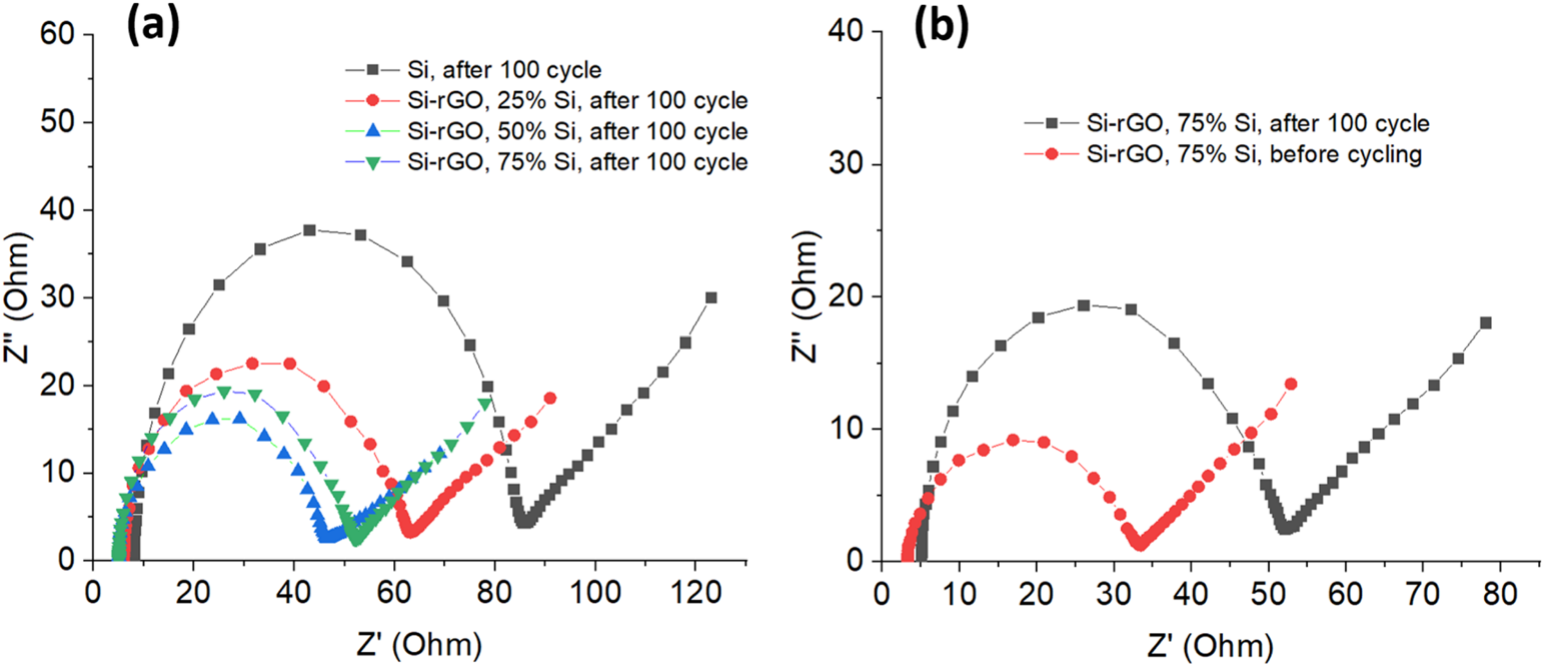
Nyquist plots of (a) pristine Si and rGO–Si composite anodes with different Si contents after 100 cycles and (b) rGO–Si (75 wt%) electrode before and after cycling.

To further elucidate the interfacial charge-transfer behavior and lithium-ion transport kinetics of the rGO–Si composite anodes, electrochemical impedance spectroscopy (EIS) measurements were performed before and after cycling under identical testing conditions (Fig. 9).

Fig. 9a shows the Nyquist plots of pristine Si and rGO–Si composite anodes with different Si contents after 100 cycles. All spectra exhibit a depressed semicircle in the high-to-medium frequency region followed by an oblique line at low frequency, indicating that the electrochemical response is governed by coupled interfacial charge-transfer and solid-state diffusion processes rather than an ideal capacitive behavior. The high-frequency intercept corresponds to the electrolyte/solution resistance, whereas the semicircle diameter is generally associated with the charge-transfer resistance at the electrode–electrolyte interface.

The pristine Si electrode displays the largest semicircle diameter after cycling, reflecting a high charge-transfer resistance at the electrode–electrolyte interface. Instead of being solely attributed to the intrinsic conductivity of Si, this large impedance can be reasonably associated with the mechanically unstable interface formed during repeated lithiation/delithiation, where particle fracture and continuous SEI reconstruction progressively disrupt electronic and ionic pathways.^2,74^ Such structural degradation is likely to leads to unstable electrical contacts and increased interfacial resistance during prolonged cycling. This interpretation is consistent with the severe agglomeration and stress accumulation inferred from the morphological analysis.

In contrast, all rGO–Si composite electrodes exhibit substantially reduced semicircle diameters, indicating improved interfacial kinetics. The presence of rGO does not merely provide a conductive additive; rather, the interconnected rGO framework is expected to provide continuous electron pathways while maintaining intimate contact with Si nanoparticles, thereby helping to suppress interfacial heterogeneity that typically dominates the impedance evolution of pure Si electrodes.^61^ In addition, the flexible rGO matrix helps buffer the large volume changes of Si during lithiation and delithiation, thereby maintaining electrical connectivity within the electrode structure. This kinetic stabilization is in line with the preserved crystallinity observed in XRD and the uniform Si dispersion revealed by SEM.

A systematic dependence of impedance behavior on Si content can be observed among the composites. The rGO–Si 75% electrode shows the smallest semicircle after 100 cycles, followed by the 50% and 25% counterparts. This observation indicates that the impedance behavior is governed not only by the amount of rGO but also by the structural configuration of the conductive network within the composite electrode. This trend is consistent with the electrochemical results, where the 75 wt% Si composite delivers the best overall performance, indicating that the optimized balance between conductive network integrity and active material loading is achieved at this composition.

From a structural perspective, the effective charge-transfer resistance in graphene-based hybrid anodes is strongly influenced by the electronic percolation pathways established throughout the electrode. When the Si fraction is relatively low (25 wt%), the graphene sheets may tend to restack more densely due to strong π–π interactions, forming compact domains that partially limit electrolyte accessibility and reduce the effective electrochemically active interface. In such a configuration, although the nominal rGO content is higher, the electron transport pathways may become locally tortuous, which may contribute to a slightly increased interfacial resistance.

By contrast, increasing the Si fraction to 50–75 wt% introduces a larger number of dispersed nanoparticles between the wrinkled graphene sheets, which effectively act as spacers that mitigate excessive restacking of the rGO layers. This structural separation helps preserve an open and interconnected conductive framework, facilitating both electron transport along the graphene network and charge transfer at the Si/electrolyte interface. As a result, the composite electrode can exhibit a lower charge-transfer resistance despite containing a smaller proportion of rGO. Similar behavior has been reported for graphene–Si hybrid systems, where the electrochemical impedance is primarily dictated by the integrity of the conductive percolation network rather than the absolute carbon content.^3,49^

It should be noted that the impedance evolution in composite electrodes does not necessarily follow a simple monotonic dependence on the carbon fraction. Instead, the measured charge-transfer resistance reflects a combined effect of electronic percolation, particle dispersion, electrode porosity, and the stability of the electrode–electrolyte interface after cycling. Therefore, moderate variations among the rGO–Si compositions are reasonable and have been widely reported in graphene–Si hybrid systems.

The low-frequency region further provides insight into Li^+^ transport behavior. The more gradually inclined Warburg tail observed for the rGO–Si composites, particularly the 75% sample, suggests facilitated ion diffusion compared with pristine Si. This behavior can be correlated with the hierarchical porous structure formed by wrinkled rGO sheets, which may shorten diffusion pathways and maintains electrolyte penetration even after repeated cycling, as suggested by the morphological observations.^15,51^

Fig. 9b compares the rGO–Si 75% electrode before and after 100 cycles. Although a moderate increase in impedance is typically observed due to SEI evolution, the absence of a dramatic enlargement of the semicircle suggests that the interfacial structure remains relatively intact. This relatively stable impedance evolution indicates that the rGO framework effectively mitigates the degradation of electrical contacts during repeated lithiation/delithiation cycles. The result is consistent with the cycling data, where capacity retention is preserved over extended cycles, suggesting that the rGO matrix moderates, rather than eliminates, interfacial degradation.^87–89^

Overall, the EIS results do not stand alone but reinforce the structural and electrochemical analyses presented earlier. By correlating reduced charge-transfer resistance and stabilized diffusion behavior with the observed structural integrity and cycling stability, the impedance spectra provide supporting evidence that the rGO–Si hybrid architecture plays a key role in governing the electrochemical performance through interfacial stabilization and kinetic regulation. These findings highlight the critical role of optimizing the conductive percolation network and structural architecture of the composite electrode, which together strongly influence the interfacial charge-transfer behavior and long-term electrochemical stability of the rGO–Si anodes.

### 3.8. Lithium storage mechanism and performance benchmarking

**Fig. 10:**
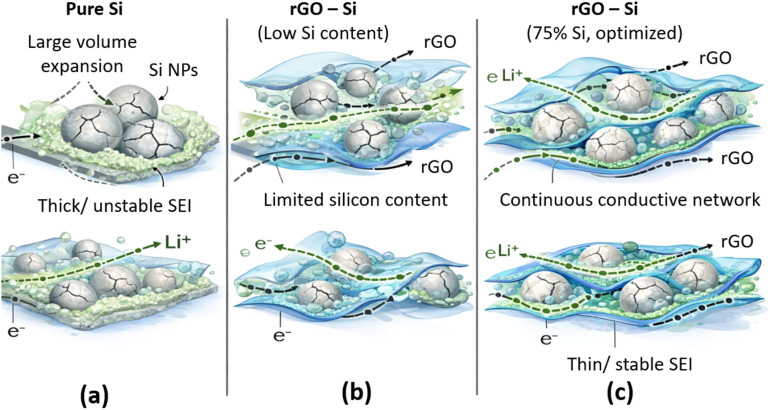
Schematic diagram of the proposed lithium storage mechanism for (a) pure Si, (b) rGO–Si composite with low Si content, and (c) optimized rGO–Si composite (75% Si).

The enhanced electrochemical performance of the rGO–Si composite anode can be attributed to the synergistic interaction between silicon nanoparticles and the reduced graphene oxide framework, as schematically illustrated in Fig. 10. In this composite architecture, silicon functions as the primary lithium-storage phase through reversible alloying and dealloying reactions with lithium, thereby providing high theoretical capacity. However, in conventional silicon anodes, the large volume variation during lithiation and delithiation induces severe mechanical stress, leading to particle pulverization, electrical isolation, and continuous instability of the solid electrolyte interphase (SEI), which collectively result in rapid capacity fading.^90,91^ As illustrated in Fig. 10a, lithium storage in silicon proceeds *via* repeated formation and decomposition of LixSi phases. Without structural confinement, these reactions generate extensive volumetric strain, causing fracture of silicon particles and disruption of electron transport pathways. In contrast, as shown in Fig. 10b, the rGO sheets in the composite form a flexible and mechanically compliant matrix that accommodates the volumetric expansion of silicon nanoparticles during lithiation. This buffering effect mitigates structural fracture and helps maintain interparticle contact throughout repeated cycling, consistent with previous reports on graphene and carbon-supported silicon architectures.^28,92^

Simultaneously, the two-dimensional rGO framework establishes a continuous conductive network, facilitating electron transport across the electrode and promoting more homogeneous electrochemical reactions. Rather than eliminating SEI formation, the presence of rGO moderates interfacial reactions by stabilizing the electrode structure, as schematically depicted in Fig. 10c. This stabilization suppresses repeated SEI rupture and reformation, contributing to improved coulombic efficiency after the initial cycles, a behavior commonly observed in carbon–silicon composite anodes with mechanically robust frameworks.^90,91^

In the composition-optimized system (75 wt% Si), the silicon content plays a critical role in balancing capacity and structural stability. As discussed in Section 3.6 and shown in Fig. 8a, the composite with 75 wt% Si achieves a favorable compromise between active material loading and mechanical buffering. Higher silicon fractions without sufficient conductive and elastic support tend to exhibit accelerated capacity decay, while lower silicon contents limit the achievable specific capacity. In addition, the interconnected rGO network shortens lithium-ion diffusion pathways and enhances charge transport, contributing to the improved rate capability observed at elevated current densities in Fig. 8b.

To further contextualize the electrochemical performance of the present rGO–Si composite, Table 1 summarizes representative silicon–carbon and silicon–graphene-based anodes reported in the literature. For example, Si/CNT/rGO composite films deliver ∼964.7 mA h g^−1^ with ∼93.8% capacity retention after 100 cycles at 0.1 A g^−1^, benefiting from conductive carbon networks and flexible architectures.^90^ Double-protected Si–CNF@C composites achieve ∼1200 mA h g^−1^ at 0.5 A g^−1^, although capacity retention decreases to ∼82% after 100 cycles due to gradual structural degradation.^91^ Yolk–shell Si@void@C structures exhibit improved cycling stability over a wide current range, but their reversible capacity varies significantly depending on structural design and operating conditions.^92^ Size-tailored Si@rGO systems further demonstrate that graphene architecture strongly influences cycling stability and rate performance.^28^

**Table 1:** Benchmark comparison of silicon–graphene/carbon composite anodes

Material system	Si content (wt%)	Current density	Reversible capacity (after ∼100 cycles)	Cycling retention	Reference
Si/CNT/rGO composite	—	0.1 A g^−1^	∼964.7 mA h g^−1^	∼93.8% after 100 cycles	90
Si–CNF@C (double protected)	—	0.5 A g^−1^	∼1200 mA h g^−1^	∼82% after 100 cycles	91
Si@void@C (yolk–shell Si–C)	—	0.1–1.0 A g^−1^	∼735–∼1238 mA h g^−1^	Retained 735 mA h g^−1^ after 100 cycles (1 A g^−1^)	92
Si@rGO (size-tailored rGO–Si)	—	∼0.2–0.5 A g^−1^	Improved retention *vs.* pure Si	Capacity retention improved	28
**This work (rGO–Si)**	**75**	**0.5 A g^−^** ^**1**^	**∼1150–1200 mA h g^−^** ^**1**^ **(100 cycles)**	**∼85–90% after 100 cycles**	**This work**

Compared with these systems, the rGO–Si composite developed in this work delivers a reversible capacity of approximately ∼1150–1200 mA h g^−1^ and maintains ∼85–90% capacity retention after 100 cycles at 0.5 A g^−1^. Notably, this performance is achieved through a relatively simple and scalable synthesis route, without relying on complex yolk–shell or hierarchical architectures. Collectively, these results highlight the importance of rational compositional and structural optimization in achieving durable, high-capacity silicon-based anodes for next-generation lithium-ion batteries.

## 4. Conclusions

In this study, binder-free rGO–Si composite anodes with systematically controlled silicon contents were fabricated *via* a simple and scalable approach to elucidate the role of composition in governing electrochemical performance. The results demonstrate that careful regulation of the Si/rGO ratio is critical for achieving a balance between high reversible capacity and structural stability during prolonged cycling. Among the investigated samples, the rGO–Si electrode containing 75 wt% Si delivers the best overall electrochemical performance, exhibiting a reversible capacity of approximately 1150 mA h g^−1^ at 0.5 A g^−1^ and retaining about 90% of its capacity after 100 cycles. Thermogravimetric analysis confirms the compositional robustness and enhanced thermal stability of Si-rich composites, supporting the effective integration of silicon nanoparticles within the rGO framework. In addition, electrochemical impedance analysis reveals reduced charge-transfer resistance and stable interfacial characteristics for the optimized electrode, consistent with its improved cycling durability. These results indicate that the interconnected and mechanically compliant rGO network not only facilitates electron transport but also effectively accommodates the volume variation of silicon during repeated lithiation/delithiation, thereby preserving electrode integrity. Overall, this work highlights that composition optimization, rather than structural complexity alone, plays a decisive role in determining the performance of rGO–Si composite anodes. The findings provide a practical guideline for the rational design of durable and high-performance silicon-based anodes for next-generation lithium-ion batteries.

## Conflicts of interest

The authors declare no possible conflict of interests.

## Supplementary Material

RA-016-D6RA01135G-s001

## Data Availability

The authors confirm that the data supporting the findings of this study are available within the articles. Raw data that support the findings of this study are available from the corresponding author, upon reasonable request. Supplementary information (SI): additional scanning electron microscopy (SEM) images of the rGO-Si composites at various magnifications and a comprehensive analysis of their multi-scale morphology, further supporting the structural interpretation and discussion presented in the main text. See DOI: https://doi.org/10.1039/d6ra01135g.
