# Supplementary material for: Binder-free rGO–Si composite anodes with controlled silicon content and composition-dependent electrochemical performance
Source: RSC Adv. 2026 Apr 8;16(20):18106–21. doi: 10.1039/d6ra01135g (PMC13060622; doi:10.1039/d6ra01135g)
Supplement: RA-016-D6RA01135G-s001 [file RA-016-D6RA01135G-s001.pdf]

Supplementary Information (SI) for RSC Advances.
This journal is © The Royal Society of Chemistry 2026

Supporting Information

Binder-free rGO-Si composite anodes with controlled silicon content and composition-dependent electrochemical performance

Pham The Tan[1], Nguyen Van Tu[2] and Nguyen Van Hao[3]*

[1] Hung Yen University of Technology and Education, Viet Tien ward, Hung Yen Province, Vietnam

[2] Institute of Materials Science, Vietnam Academy of Science and Technology, Hanoi, Vietnam

[3] TNU-University of Sciences, Phan Dinh Phung ward, Thai Nguyen Province, Vietnam

*Corresponding author: haonv@tnus.edu.vn

S1. Additional SEM analysis at different magnifications

To further elucidate the morphological features of the rGO-Si composites, additional scanning electron microscopy (SEM) images at different magnifications are presented in Figures S1-S3. These images complement the representative SEM observations shown in the main manuscript (Fig. 4a-c) and provide a more comprehensive view of the structural characteristics across multiple length scales.

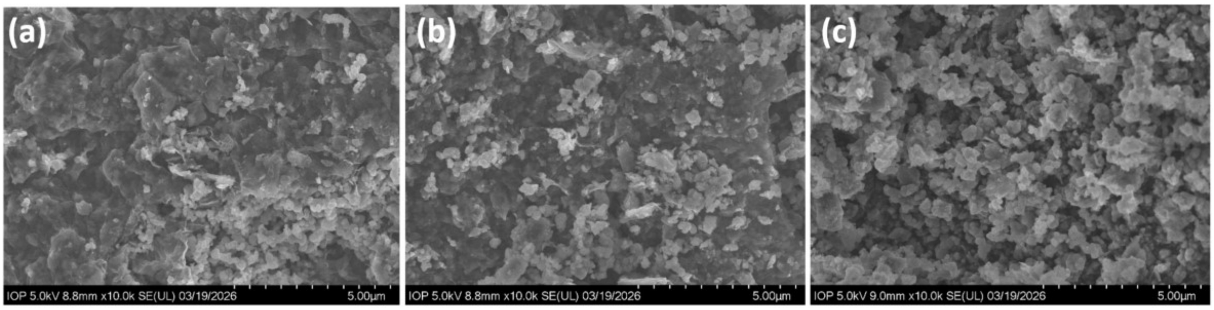

Figure S1. SEM images of rGO-Si composites at low magnification (~5 µm scale) for different Si contents: (a) 25 wt%, (b) 50 wt%, and (c) 75 wt%.

At this scale, all samples exhibit a continuous and interconnected graphene network extending over micrometer dimensions. The wrinkled morphology of rGO sheets is clearly visible, forming a porous framework. The overall structural integrity of the network is maintained across all compositions; however, differences in particle distribution can already be observed. The 25 wt% sample shows a more compact graphene structure, while the 75 wt% sample

displays more pronounced particle clustering, indicating increased aggregation at higher silicon content.

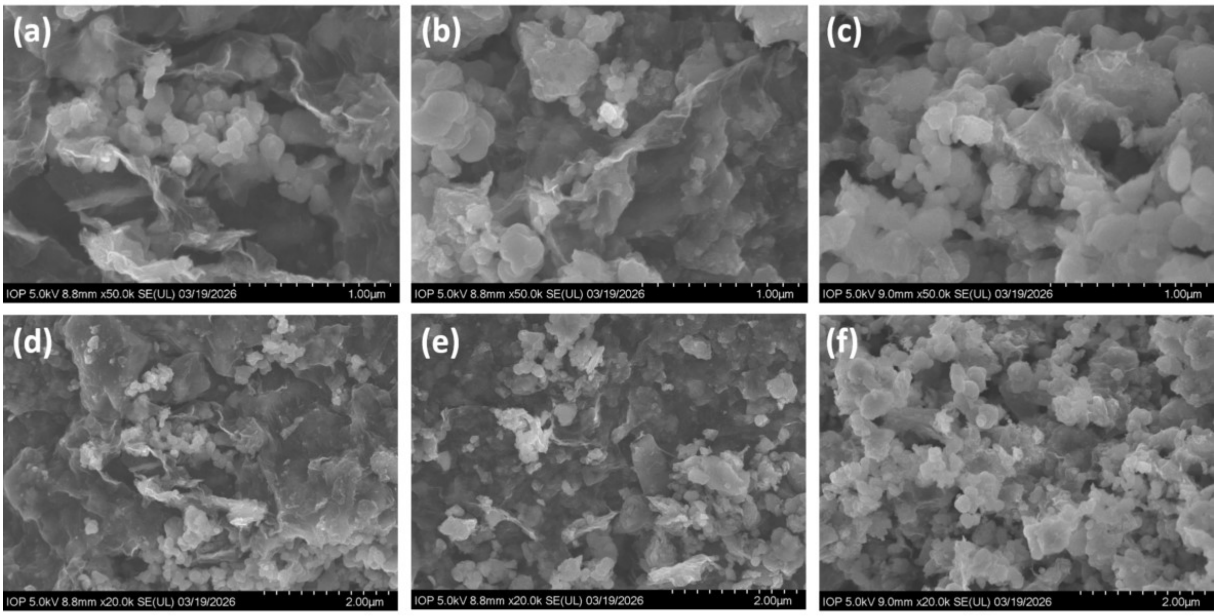

Figure S2. SEM images of rGO-Si composites at intermediate magnification (~1-2 μm scale): (a,d) 25 wt%, (b,e) 50 wt%, and (c,f) 75 wt%.

These images correspond to the characteristic length scale used in the main text (Fig. 4a-c) and provide additional confirmation of the composition-dependent morphology. Silicon nanoparticles can be more clearly distinguished within the rGO framework. The 50 wt% sample shows a relatively homogeneous distribution with improved spacing between graphene sheets, whereas the 75 wt% sample exhibits more frequent particle aggregation and reduced uniformity.

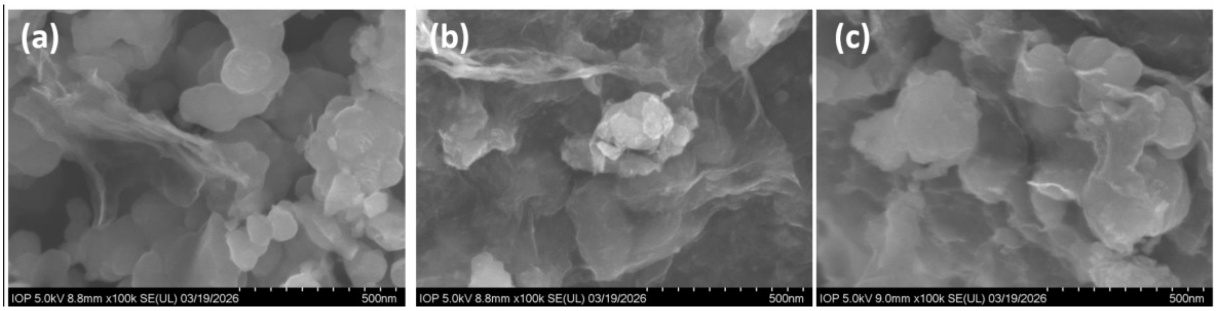

Figure S3. High-magnification SEM images (~500 nm scale) of rGO-Si composites acquired from different regions of the samples: (a) 25 wt%, (b) 50 wt%, and (c) 75 wt%. These images provide additional evidence of the local morphological variation and confirm the representative nature of the SEM images shown in the main text (Fig. 4a-c).

At higher magnification, the nanoscale features of the composites become evident. Silicon nanoparticles are observed as discrete clusters embedded within or attached to the rGO sheets.

The images acquired from different regions confirm that the morphological features observed in the main manuscript (Fig. 4) are representative and reproducible across the sample. While minor local variations in particle distribution are present, the overall structural characteristics remain consistent.

S2. Discussion of multi-scale morphology

The combined SEM observations across different magnifications (Figs. S1-S3) confirm that the rGO-Si composites possess a hierarchical structure spanning from the nanoscale to the microscale. At the microscale, a continuous and interconnected graphene network is formed, providing structural integrity and conductive pathways. At the nanoscale, silicon nanoparticles are distributed within this network, although partial aggregation is observed, particularly at higher silicon content.

These multi-scale structural features support the interpretation presented in the main manuscript, where the rGO framework acts as a conductive and mechanically flexible matrix, while the distribution of silicon nanoparticles influences the electrochemical performance. The consistency of morphological characteristics across different magnifications further validates the reliability of the structural analysis.
